# Lymphopenia in Cancer Patients and its Effects on Response to Immunotherapy: an opportunity for combination with Cytokines?

**DOI:** 10.1186/s40425-019-0549-5

**Published:** 2019-03-28

**Authors:** Christine Ménétrier-Caux, Isabelle Ray-Coquard, Jean-Yves Blay, Christophe Caux

**Affiliations:** 1 Univ Lyon, Université Claude Bernard Lyon 1, INSERM 1052, CNRS 5286, Cancer Research Center of Lyon (CRCL), Centre Léon Bérard, F-69008 Lyon, France; 2 Innovation in Immuno-monitoring and Immunotherapy Platform (PI3), Centre Léon Bérard, F-69008 Lyon, France; 3 Medical Oncology department, Centre Léon Bérard, F-69008 Lyon, France

**Keywords:** Lymphopenia, solid tumors, TCR diversity, anti-cancer immunotherapy

## Abstract

Quantitative lymphocyte alterations are frequent in patients with cancer, and strongly impact prognosis and survival. The development of cancers in immunosuppressed patients has demonstrated the contribution of different T cell populations, including CD4^+^ cells, in the control of cancer occurrence.

Whereas absolute numbers of neutrophils, platelets and red blood cells are routinely monitored in clinic following treatments, because of possible short-term complications, absolute lymphocyte counts (ALC), their subpopulations or diversity (phenotype, TCR) are rarely analyzed and never used to choose therapy or as prognostic criteria. The recent identification of immune checkpoint inhibitors (ICPi) as powerful therapeutic agents has revitalized immunotherapy of cancer in a broader group of diseases than anticipated. The status of the immune system is now recognized as an important biomarker for response to these novel treatments. Blood ALC values, along with tumor infiltration by CD8^+^T cells, and ICPi and ICPi-ligand expression, are likely to be a potential marker of sensitivity to anti-ICPi therapy.

In this article, we review the current knowledge on the incidence and significance of lymphopenia in cancer patients, and discuss therapeutic strategies to restore lymphocyte numbers.

## Introduction

The progression of solid tumors is associated with a variety of strategies developed by cancer cells to escape the immune response (for review [1]:). The development of treatments with antibodies (mAb) targeting ICPi constituted an important breakthrough leading to increased patient survival in several cancers such as melanoma, lung, kidney or bladder. ICPi blockade involves mAbs targeted against cytotoxic T-lymphocyte associated protein 4 (CTLA-4), programmed death receptor 1 (PD-1) or its ligand (PD-L1) which have all been demonstrated to inhibit T cell activation and favor T cell exhaustion or the development of regulatory T cells (Tregs). The use of ICPi blockers modulate the interaction between T lymphocytes and tumor cells or macrophages, thereby favoring the re-induction of the “natural” function of T cell populations in the tumor environment leading to a durable clinical response. However, even in cancers where these ICPi demonstrated an overall survival (OS) benefit, only 20 to 40% of patients will benefit from treatment, hence highlighting the need to identify strategies to improve efficacy. As lymphocytes have been recognized as major actors in the fight against tumor progression in several human tumors, we review herein the role of lymphopenia as a surrogate marker of initial resistance to immunotherapy and discuss therapeutic strategies to restore lymphocyte numbers and to increase the response rate of patients treated with ICPi mAbs.

## Tumor cells induce immune system dysfunctions

In murine models and in humans, the risk of tumor development is increased in the context of immunosuppression [2, 3] demonstrating the concept of tumor immuno-surveillance wherein the immune system is involved in controlling or eliminating transformed cells. Alterations in anti-tumor immune responses have been widely described since the 1980’s.

### Immune infiltrates in primary tumors

A recent meta-analysis of 20 different cancer types has established that the density and the composition of the immune infiltrate are important for tumor progression [4].

A high density of CD8^+^ T effectors in most tumors correlates with longer disease free survival (DFS) and OS [4]. In contrast, the accumulation of myeloid-derived suppressor cells (MDSC) [5], type-2 macrophages (M2-MΦ) [6]) or Tregs (for review [7]), as well as the production of suppressive cytokines and metabolites favor an immunosuppressive environment and tumor progression.

Among these suppressive cells, Tregs are selectively recruited in the tumor environment through the CCL22/CCR4 axis [8–10]. Compared to other T cells, Tregs have an activated phenotype (ICOS, GARP, CD39, GITR, HLA-DR) [9, 11–13] and expand *in situ* through ICOS/ICOS-L interaction with tumor-infiltrating plasmacytoïd dendritic cells or tumor cells [11, 13–17].

### Quantitative and functional alterations in the peripheral blood

The existence of peripheral immune alterations in cancer patients was shown for the first time in the mid-1970’s by Bone and Lauder [18] in gastrointestinal tumors. Lymphopenia has been observed in more than 20% of patients with advanced disease and only 3% with localized disease [19–21] in several tumor types (pancreas, melanoma, Non-Hodgkin’s lymphoma (NHL), breast cancer (BC), sarcomas). Moreover, an increased number of circulating neutrophils, a hallmark of inflammation, is observed in patients with solid tumors and is combined with an increased neutrophil-to-lymphocyte ratio (NLR) (for review [22]:).

Lymphopenia may affect all or only some of the T or B lymphocyte subpopulations. CD4^+^ lymphopenia is key in the clinical evolution of HIV patients [23–25], is common in many advanced cancer patients with pancreatic cancer, melanoma, NHL, BC, sarcomas or hepatocellular carcinoma (HCC) [19–21].

Furthermore, modulation of other blood subpopulations have been described such as increased frequency of Tregs (for review [7, 26]), Th17 cells [27], MDSC [28], or PD-L1^+^ T cells [29]. Most of these alterations were associated with poor prognosis [26, 30], but are not directly correlated with lymphopenia.

While CD4 lymphopenia is mostly detected in advanced or metastatic stages, functional impairment of immune cells (NK, monocytes, memory CD4^+^ and CD8^+^ T cells) can be detected in patients with localized primary tumors (BC, colon carcinoma, HCC) [31–35]. Primary tumor-derived factors alter blood monocytes that are unable to differentiate into M1-MΦ (Ramos submitted) or functional Mo-DC [36–38].

### Clonality, diversity and magnitude of the adaptive immune response

Each T cell expresses a TCR allowing its specific activation by a unique antigen presented in the context of the major histocompatibility (MHC) complex. Thus, T cell populations must express a broad polyclonal TCR repertoire to confer immune protection against infectious agents and malignant cells [39]. Recent evidences indicate that somatic mutations are the basis for the generation of potential neo-antigens recognized by tumor-infiltrating T lymphocytes (TIL) [40, 41]. A strong TCR diversity is required to generate a response against neo-epitopes and recent studies [42–48] suggest that broadening of the TCR repertoire diversity could favor tumor control.

Since the 1990’s, PCR-based technologies enabled the quantification of TCR diversity at the mRNA and genomic levels. Numerous data have reported a restriction of the TCR diversity with the appearance of an oligoclonality in TILs in comparison to peripheral T lymphocytes (for review [49]). In metastatic BC patients, peripheral blood TCR diversity is not homogenously represented and diversity is significantly reduced in comparison to healthy donors [50] but not necessarily associated with lymphopenia, thereby demonstrating the importance of combined scores to characterize T cell alterations [50].

### Lymphopenia is associated with increased cancer incidence

A meta-analysis performed in two immuno-compromised patient populations (HIV-infected and transplanted patients) [51] have shown a higher incidence of cancers due to infectious or viral causes. Other studies [52, 53] in transplanted patients reported a higher incidence of virus-induced cancers (Kaposi's sarcoma, NHL and HL) as well as tumors without established viral etiology such as head and neck carcinomas and melanomas. Moreover, CD4^+^ T cell lymphopenia in patients with Sjögren's autoimmune syndrome [54] or idiopathic CD4^+^ lymphopenia [55, 56] is associated with an increased risk of cancer. Accordingly, the restauration of immune functions in AIDS patients thanks to highly active anti-retroviral therapies (HAART) was associated with a strong reduction in the incidence and the progression of these cancers [57, 58].

Immune deficiency is therefore consistently associated to increased frequency of certain cancer types.

## Impact of lymphopenia on tumor evolution

### Lymphopenia is correlated with patient survival and toxicity of chemotherapy

Lymphopenia observed in advanced disease [19–21, 50, 59–64] correlates with patients’ performance status (PS) as well as with specific unfavorable prognostic factors [65].

Studies performed by our group and others, including over 3,000 patients with advanced cancers, have shown that regardless of the histological subtype and treatment, global and CD4^+^ lymphopenia are powerful independent predictors of risk of high grade toxicity associated with chemotherapy including febrile neutropenia (FN) [59, 64, 66, 67], severe thrombocytopenia requiring platelet transfusion [60], severe anemia requiring red blood cells (RBC) transfusion [61, 62] and increased risk of early death after chemotherapy [19–21, 63].

Randomized clinical trials have demonstrated the ability of prophylactic recombinant G-CSF (Filgrastim, ELYPSE-2) and EPOα (ELYPSE-4) to reduce FN and RBC transfusion requirements, respectively [68] in these high risk lymphopenic patients.

### Prognostic Value of lymphopenia for PFS and OS

A peripheral lymphopenia impacts on patient PFS and OS in many advanced solid tumors (Table 1). In breast and ovarian carcinomas, NHL, DLBCL, T-cell lymphomas (PTCL, PTCLU), follicular lymphoma [69, 70], sarcomas and colon carcinoma, a global (<1000/mm^3^) or severe (<700/mm^3^) lymphopenia detected before treatment [65, 66, 71–74, 88–90, 92–94], early after chemotherapy treatment (5 or 15 days) [19, 75, 91] or after radiotherapy (RT) (for review [76]:) is associated with a worse PFS and OS.

**Table 1:** Different published studies exploring the impact of the global lymphopenia or NK and T cell subsets on relapse-free survival (RFS) or overall survival (OS) in patients with solid tumors

Tumor Type	N	Type of lymphopenia evlauated	Lymphocyte Threshold (% lymphopenia)	RFS (Cox Analysis)			OS (Cox Analysis)			References
				RR	IC 95%	P value	RR	IC 95%	P value	
Sarcoma	193	Overall Lymphopenia	<1000 (24%)	Not evaluated			1.46	1.0-2.1	0.05	[68]
Ewing Sarcoma	24	Overall Lymphopenia	<500 (33%)	Not evaluated			4.34	1.35-14.28	0.007	[75]
Renal Cell Carcinoma	424	Overall Lymphopenia	≤1300 (28.06%)	Not evaluated			1.75	1.14-2.67	0.0102	[65]
Colon Carcinoma	260	Overall Lymphopenia	<1000 (19%)	1.56	1.0-2.43	0.048	2.35	2.34-4.14	0.003	[66]
Breast Carcinoma	195	Overall Lymphopenia	<1000 (28.7%)	1.82	1.27-2.59	0.001	2.23	1.36-3.65	0.001	[89]
Non Hodgkin Lymphoma	322	Overall Lymphopenia	<1000 (25%)	1.71	1.2-2.4	0.002	1.48	1.03-2.21	0.04	[68]
Diffuse large B cell lymphoma (DLBCL)	151	Overall Lymphopenia	≤1000 (35.8%)	Not evaluated			2.38	1.29-4.34	0.005	[90]
DLBCL	221	Overall Lymphopenia	<1000 (38.9%)	2.72	1.61-4.60	<0.001	2.51	1.38-4.58	0.003	[80]
DLBCL	89	Overall Lymphopenia	<840 (23%)	3.81	1.72-8.42	0.0009	4.38	1.88-13.28	0.0012	[79]
Follicular Lymphoma	228	Overall Lymphopenia	≤1000 (28%)	Not evaluated			1.72	1.33-2.24	<10^-4^	[70]
Hodgkin Lymphoma	476	Overall Lymphopenia	<600 (18.06%)	1.59	0.96-2.58	0.06	1.25	0.74-2.15	0.4	[82]
Hodgkin Lymphoma	2497	Overall Lymphopenia	<600 (11%)	1.38		0.002	Not evaluated			[81]
Multiple Myeloma	537	Overall Lymphopenia	<1400 (62%)	Not evaluated			1.71	1.53-2.35	<10^-4^	[92]
ATLL	60	Overall Lymphopenia	<1000 (35.6%)	1.93		0.004	2.37		0.0003	[93]
PTCLU	69	Overall Lymphopenia	<1000 (38%)	Not evaluated			4.0	1.9-8.3	<10^-4^	[71]
PTCL-NOS	118	Overall Lymphopenia	1000 (30.5%)	1.94	1.19-3.18	0.008	2.24	1.33-3.78	0.002	[72]
Breast Carcinoma	287	Overall Lymphopenia	<1000 (27%)	1.48	1.1-2.0	0.01	1.8	1.3-2.4	0.0002	[68]
Breast Carcinoma	195	Overall Lymphopenia	<1000 (28.7%)	1.82	1.27-2.59	0.001	2.23	1.36-3.65	0.001	[89]
Breast Carcinoma 1st relapse	128	Overall Lymphopenia	<1000 (44.27%)	Not evaluated			1.8	1.15-2.82	0.01	[50]^b^
Breast Carcinoma 1st relapse 1^st^ relapse	103	Overall Lymphopenia	<700 (22.3%)	Not evaluated			2.03	1.17-3.50	0.016	[21]^b^
Breast Carcinoma 1st relapse 1^st^ relapse	103	CD4^+^ Lymphopenia	≤450 (53.4%)	Not evaluated			2.50	1.57-3.98	<10^-4^	[21]^b^
Breast Carcinoma >2^nd^ relapse	101	CD4^+^ Lymphopenia	≤450 (70.3%)	1.35	0.87-1.1	0.183	1.69	1.04-2.78	0.036	[21]
Metastatic Solid Tumors	219	CD4^+^ Lymphopenia	≤450 (47.9%)	Not evaluated			1.5	1.1-2.1	0.017	[20]
Metastatic Solid Tumors	213	CD4^+^ Lymphopenia	<450 (49.7%)	Not evaluated			7.7^a^	1.6-35^a^	0.007^a^	[19]^a^
Non Hodgkin Lymphoma	88	CD8^+^ Lymphopenia	<200	Not evaluated			3.30	1.21-9.0	0.01	[88]
Follicular Lymphoma	75	NK cells Lymphopenia	<150 (44%)	Not evaluated			6.73	0.76-59	0.08	[69]
DLBCL	136	NK cells Lymphopenia	≤80 (37.5%)	1.81	1.27-2.57	0.001	Not evaluated			[94]

^a^ Analysis of the risk of early death; ^b^ Univariate analysis only

Other studies using different thresholds of lymphopenia from moderate (<1500/mm^3^) to severe (<600/mm^3^), report similar results in recurrent desmoid tumors [77], pancreatic tumors [78], renal cell carcinoma (RCC) [65], diffuse large B cell lymphomas (DLBCL) [79, 80], Hodgkin’s disease [81, 82], and HCC [83].

Scores combining different biological parameters have also been evaluated for their capacity to predict outcome. Inflammatory markers, such as the NLR, the monocyte-to-lymphocyte ratio (MLR), and the platelet-to-lymphocyte ratio (PLR), used as markers of systemic inflammation, are associated with poor outcomes in several malignancies [84, 85]. An elevated NLR, which reflects a combination of increased number of neutrophils and/or lymphopenia, is associated with worse outcome in many solid tumors, both in early and advanced stages [85]. Moreover, a high serum lactate dehydrogenase level (LDH)-to-ALC ratio predicts a poor intra-tumoral immune response and reduced survival in DLBCL patients [86].

Altogether, lymphopenia is consistently reported as an adverse prognostic factor for PFS or OS in a variety of tumors. In first line metastatic BC patients [50] patients with both lymphopenia and low TCR diversity (16.4% of the cohort) before any treatment have an increased risk of early death after chemotherapy [50]. All lymphocyte compartments are quantitatively altered [21]. While NK cell and CD8^+^ T cell or B cell lymphopenia correlate poorly with OS, CD4^+^ lymphopenia and severe lymphopenia (<200/mm^3^) strongly impact patient survival with respectively 40% and 90% reduction of the median OS or PFS. In multivariate analyses, CD4^+^ lymphopenia is an independent poor prognosis factor for OS in several cohorts of advanced BC (1^st^ line and > 2^nd^ line) [21], and other solid tumors (NHL, myelomas and sarcomas) [20]. Furthermore in Ewing sarcoma [75] and osteosarcoma patients [87], early ALC recovery after chemotherapy treatment predicts better outcome. Conversely in NHL, altered CD8^+^ T cell numbers (<200/mm^3^) correlate with a poor prognosis [88] whereas CD4^+^ lymphopenia (<500/mm^3^) does not. This might be related to the role of CD4^+^ T cells as helpers for B cell tumor development.

Altogether, these data demonstrate the negative prognostic value of CD4^+^ lymphopenia and its significance as a predictor of non-response to chemotherapy suggesting the important role of CD4^+^ T cells in controlling tumor progression and/or development of opportunistic infections in cancer patients.

### Lymphopenia is also favored by radiotherapy, chemotherapy and targeted therapies

Although, localized RT treatment could favor anti-tumor immunity through abscopal effects [95], RT also strongly favors lymphopenia (for review [76]). Indeed, in addition to affecting TILs, RT also negatively impacts circulating lymphocytes transiting through the irradiated field favoring lymphopenia. In this context, RT of tumors near lymphopoïetic sites will also contribute to lymphopenia. Moreover, RT-associated lymphopenia depends on the radiation modality with RT-target volume playing an important role [96]. The reduction of radiation doses using either proton beam therapy (PBT) that spares surrounding tissue from radiation or reduction of the duration of exposure using either short course radiation with stereotactic body RT (SBRT) or hypo-fractionated schedule will mitigate normal tissue exposure reducing the risk of severe lymphopenia [97, 98]. Additionally the impact of RT also depends on the environment since a cancer-related inflammatory state, as shown by neutrophilia, before radiation is correlated with an increased susceptibility to RT-induced grade-4 lymphopenia [99].

Cyclophosphamide, cisplatin, methotrexate, and taxanes are among the most powerful chemotherapies inducing lymphopenia. Treatment of hematological tumors with fludarabine strongly reduces CTL numbers [100, 101]. Of interest, metronomic treatment with cyclophosphamide has proven its capacity to limit lymphopenia by selectively damaging Tregs [102], thus mitigating immunosuppression and promoting anti-tumor T cell immunity.

Several therapies targeting oncogenic signaling pathways or molecular abnormalities also induce lymphopenia, including all patients receiving mTOR inhibitors [103] and 20% of patients treated with tyrosine-kinase receptor inhibitors targeting VEGFR or PDGFR [104]. Intriguingly, whereas MEK inhibitors do not alter ALC, among BRAF inhibitor treatments, vemurafenib [105] but not dabrafenib [105, 106] induces lymphopenia affecting principally CD4^+^ T cells that may either result from a change in their compartmental distribution in the tumor [107] or secondary lymphoid tissue or from an absolute lymphopenia due to direct effect of BRAF inhibition on T cells.

The combination of chemotherapy and immunotherapy may also induce severe lymphopenia. For example, the treatment of DLBCL by the combination of an alkylating agent, and an anti-CD20 antibody, induces a CD4^+^ T cell lymphopenia in 66% of cases, that is reversed upon treatment discontinuation while anti-CD20-induced B cell lymphopenia persists [108].

Several studies have shown that naïve CD4^+^ T cell regeneration requires an active thymic function present in children, and is less effective in adults and elderly patients while CD8^+^ T cell recovery is faster and less dependent on thymic activity [109–111].

## Putative mechanisms contributing to cancer patient lymphopenia

Several mechanisms may account for lymphopenia detected in metastatic cancer patients; i) destruction of lymphocytes induced by tumor cells expressing pro-apoptotic ligands [112], ii) reduced capacity of lymphocytes to respond to TCR stimulation due to an altered expression of the ζ chain of the TCR [113], iii) a high proportion of CTLA-4-expressing Tregs favoring immunosuppression [114] or iv) activation induced cell death (AICD) [115]. In the tumor environment, PDL-1 expressed on tumor cells or macrophages (for review [116]) may lead to reduced TIL proliferation and survival [117] that could have a systemic impact.

However, the reduction of naive CD4^+^ T cell numbers suggests an alteration of the generation of new naive CD4^+^ T lymphocytes, which may result from i) a reduced thymic function, ii) a defect in production of homeostatic cytokines (IL-7, IL-15, IL-2) or of their receptors (IL-7Rα) or iii) an impairment in recruitment of cells generated in the thymus to the periphery *via* a reduction in plasma levels of chemokines (CCL22) [21].

It remains to be determined whether lymphopenia is associated with somatic tumor characteristics or to constitutional patient characteristics, or both.

## Therapies to restore a physiological number of lymphocytes in the peripheral blood

The identification of treatments able to restore a physiological number of T cells is of major importance. Different immunotherapy treatments have been evaluated in HIV “immune non-responders” patients, but also in patients with virus-induced (HCV) chronic hepatitis, idiopathic CD4^+^ lymphopenia, and hematological malignancies treated with HSCT, or in chemotherapy-treated advanced cancer patients.

IL-2, IL-7 and IL-15 are three cytokines playing a role in the development, proliferation and survival of T cells that have been evaluated to restore lymphopenia. IL-2 binds to its high affinity receptor composed of three subunits including IL-2Rα (CD25), IL-2Rβ (CD122), and IL-2 receptor γ chain (CD132) common to multiple cytokines (IL-2, IL-4, IL-15, IL-7, IL-9 and IL-21). IL-15 signals through a hetero-trimeric receptor involving CD132 and CD122 shared with IL-2 and a third receptor subunit, specific for IL-15 (IL-15Rα). IL-7 signals through a heterodimer that consists of two chains: IL-7Rα (CD127), shared with thymic stromal lymphopoietin (TSLP), and CD132.

### Interleukin-2 (IL-2)

IL-2, a key cytokine responsible for both CD4^+^ and CD8^+^ T cell proliferation (for review [118]), has been evaluated in HIV patients with severe CD4^+^ lymphopenia. However, despite a marked increase in absolute CD4^+^ T cell number, no clinical benefit was observed [119, 120]. The proportion of Tregs among CD4^+^ T cells [120] was increased as i) IL-2 delivers signals essential for thymic or peripheral Tregs differentiation [121] and ii) Tregs expressing high CD25 levels have a competitive advantage over other T cell subpopulations [122].

The injection of high doses of IL-2, one of the first immunotherapies approved by the FDA as a single agent for treatment of metastatic melanoma and RCC, induced significant hematological toxicities including thrombocytopenia and lymphopenia [123, 124]. Increased perforin^+^ NK cells and activated CD8^+^ T cell numbers were described in the periphery and within the tumor mass in most treated patients demonstrating an activation of the anti-tumor immune response, but this remained transient and was not always correlated with tumor regression or clinical response [125] probably due to the strong concomitant amplification of Tregs [126, 127].

The design of mutant IL-2 molecules (IL-2v) provides an alternative for improving the biological activity of IL-2. Several groups have designed IL-2v that bind conventionally on CD122/CD132 but have reduced affinity to IL-2Rα which prevents Tregs expansion [128–130].

### Interleukin-15 (IL-15)

IL-15 shares structural similarities with IL-2 and plays a central role in controlling the life and death of NK cells  and memory CD8^+^ T cells (for review [131]). Although IL-15 and IL-2 share a number of functions on T cell subpopulations, B cells and NK cells, in many adaptive immune responses, they have distinct and often conflicting effects. Unlike IL-2, IL-15 is not required for the maintenance of Tregs and in contrast to IL-2 that promotes AICD, IL-15 reduces apoptosis of activated T cells [132]. IL-15 promotes memory CD8^+^ T cells survival and plays a major role in maintaining high affinity and sustainable T cell responses against pathogens [133].

These functional differences may partly result from the source of cytokine production since IL-2 is produced by T cells, NK and NKT cells whereas IL-15 is mainly produced by stromal cells, DC, monocytes/macrophages and endothelial cells. Additionally, these cytokines differ in terms of mode of action, since IL-15 associated to IL-15Rα at the surface of a producer cell interacts with CD122/CD132 on T cells to deliver an activation signal at the immunological synapse by trans-presentation. On the other hand, IL-2, which is secreted, has more distant and bystander biological effects. Ten days treatment of macaques rhesus with IL-15 strongly increased NK cell numbers and induced a huge amplification of circulating effector-memory CD8^+^ T cells [134] whereas memory CD4^+^ T cells were less affected and Tregs underwent weak stimulation [135].

Several ongoing clinical trials are evaluating the impact of rhIL-15 in advanced cancer patients. A first pilot study reported the capacity of rhIL-15 to drastically increase NK cells, Tγδ and CD8^+^ T cell numbers that return to baseline two weeks after treatment completion [136]. However IL-15 treatment causes fever and reduced blood pressure [137], while also inducing the expression of ICPi such as PD-1 and the immunosuppressive cytokine IL-10 secreted by CD8^+^ T cells [138]. The IL-15 super-agonist (ALT-803), a (N72D) IL-15/IL-15Rα/IgG1-Fc complex with potent agonist function to the CD122/CD132 dimers [139] and increased half-life [140] is currently being evaluated in clinical trials in ovarian cancer (NCT03054909), multiple myeloma (NCT02099539) and AML (NCT02989844) without any efficacy result published yet.

### Interleukin-7 (IL-7)

IL-7 is a non-redundant cytokine secreted by fetal liver cells, bone marrow and thymic stromal cells, as well as epithelial cells (enterocytes, keratinocytes) and follicular dendritic cells (for review [141]). IL-7 is essential for T cell generation as IL-7- or IL-7R-deficient mice as well as IL-7Rα mutant patients with severe combined immunodeficiency (SCID) do not generate mature T lymphocytes [142, 143]. In these situations, thymocytes are blocked at an early stage of differentiation and thymic cellularity is considerably reduced [144]. Moreover, IL-7 is a key player in the regulation of peripheral T cell homeostasis [145] and is involved in survival, proliferation, differentiation, and metabolism of peripheral T lymphocytes [146]. Thus IL-7 is a potent therapeutic candidate for immune reconstitution.

IL-7 has been successfully tested in various immune reconstitution models. In mouse models of chemotherapy-induced lymphopenia, IL-7 accelerates CD4^+^ and CD8^+^ T cell repopulation in spleen and lymph nodes [147]. An immune reconstitution is observed in murine and baboon allogeneic HSCT models treated with IL-7 [148, 149] and has recently been evaluated to counteract IFNα-induced lymphopenia [150] and treat SIV infected monkeys [151]. In the preclinical models evaluated, IL-7 injection does not induce the acute inflammatory cytokine release observed with IL-2 [141]. In preclinical tumor models, IL-7 alone does not allow the development of anti-tumor immune response yet amplifies a response induced by vaccination or adoptive T cell transfer [152].

rhIL-7 has been evaluated in the clinic for the treatment of severe lymphopenia in “immune non-responder” HIV patients, post-HSCT transplanted patients, patients with idiopathic lymphopenia, and advanced cancer patients post-chemotherapy and has demonstrated an effect on T lymphocytes in terms of increased cell counts, quality and TCR diversity [44–48]. To date, fifteen clinical trials evaluating the subcutaneous injection of rhIL-7 with variable dosing schedules have been recorded worldwide. So far, the clinical data show that rhIL-7 is well tolerated (for review [153, 154]).

Treatment is associated with early but transient T cell depletion that could result from a rapid migration of T cells from the blood to various organs (glands, intestine, skin) as shown in monkey studies [155]. This period is followed by a peripheral CD4^+^ and CD8^+^ T cells expansion in all treated patients [44–48] for a long period (> 10 months).

rhIL-7 injections increase memory but also naive T cells [47] that partly result from the blood margination of recent thymic emigrants (CD31^high^TREC^+^) reflecting thymic reactivation. CD4^+^ T cells generated are conventional T cells with a detectable helper function and Treg counts are decreased [156]. Treatment with rhIL-7 also promotes the generation of T_EM_ and T_CM_, of importance for a long-term protection [47] and amplifies T cell diversity [157, 158], including in advanced cancer patients [159].

The Phase-2a ELYPSE-7 clinical trial (NCT01368107), a double-blind study randomizing recombinant IL-7 (CYT107) against placebo promoted by our institution in collaboration with Cytheris demonstrated that one cycle of CYT107 partially restored T cell pools in lymphopenic cancer patients, increasing both naïve and memory CD4^+^ and CD8^+^ subsets without altering their functional capacities [160] even during chemotherapy treatment. This effect was transient over time thus repeated CYT107 are required to induce a sustained increase in T cell subsets as reported by Thiébaut *et al*. in HIV-infected patients, underlining the importance of repeated cycles of IL-7 for long-term CD4^+^ T cell response [161]. More recently, a NCI study (NCT00923351) reported restoration of CD4^+^ T cell counts by CYT107 treatment in pediatric sarcoma patients treated by a combination of standard antineoplastic therapy followed by autologous lymphocytes and tumor lysate/KLH-pulsed dendritic cell vaccinations [162].

Overall, IL-7 is expected to broaden the amplitude of the response against malignant cells that escape the immune system by mutation and immuno-editing and should help metastatic cancer patients to restore TCR repertoire diversity for improved anti-tumor immune response.

## Lymphopenia, other peripheral biomarkers and ICPi treatment

Whereas, modulation of immune cell composition in solid tumor environment during anti ICPi treatment has been widely described (for review [163]), the changes in periphery remain less well characterized. Retrospective peripheral blood immuno-monitoring studies were conducted on small cohorts to determine if the number and quality of circulating immune cells might affect their efficacy.

### Lymphopenia and other T cell biomarkers

- **Lymphopenia**

ICPi (PD-1, TIM-3, CTLA-4) expression by TILs in solid tumors known to be involved in the inhibition of T cell activation/proliferation could participate to peripheral lymphopenia. The therapeutic activity of anti-CTLA-4 and anti-PD-1/L1 Abs (for review [164]) may, in part, act through the restoration of normal numbers of circulating lymphocytes.

Interestingly, baseline lymphopenia [165] or increased ALC 2 to 8 weeks [166–168] after the first ipilimumab injection have been reported as possible biomarker candidates for improved outcome. Moreover, in advanced melanoma patients with severe lymphopenia, tremelimumab rapidly restores the effector and memory CD4^+^ and CD8^+^ T-cell pool and TCR-dependent T-cell proliferation whereas NK cell lymphopenia is not affected [169].

Low ALC also impacts efficacy of anti-PD-1/ -L1 as lymphopenia is a worse prognosis factor both at baseline [170–172] and after two to three months of treatment [170, 172].

- **T cell subsets**

T cells are the main effector cells of ICPi treatment. Retrospective analysis of blood T cell subsets in anti-CTLA-4-treated melanoma patients revealed that a high proportion of Tregs before treatment was associated with better survival [166, 173]. Their constitutive expression of CTLA-4 could make them the preferred targets of ipilimumab. Anti-CTLA-4 efficacy has also been associated with reductions in naïve T cells [174] and increase in melanoma-reactive CD8^+^ cytotoxic T cells [175]. Finally, ipilimumab treatment resulted in consistently durable (3- and 6- months) increased proliferation of both CD4^+^ and CD8^+^ T cells [176]. Likewise, screening of anti-PD-1 treated melanoma patients’ blood by high dimensional clustering, identified the increase of a subset of CD4^+^ T_CM_ in long-term survivors, but not in non-responders [177].

- **Expression of ICP/ICP-L on T cells**

High PDL-1 expression on T cells (CD4^+^ and CD8^+^) was associated with worse PFS and OS after anti-CTLA-4 treatment of melanoma [30] and might, therefore, be a mechanism for tumor immune escape to anti-CTLA-4 treatment. Increased blood frequency of ICOS-expressing CD4^+^ and CD8^+^ T cells which comprise a population of effector T cells [178–181] is considered as a reliable biomarker of clinical response to anti-CTLA-4. Moreover, detection in blood of high risk resected melanoma of CD137-expressing CD8^+^ T cells, that could relate to tumor antigen-specific T cells, was significantly associated with a lack of relapse after adjuvant ipilimumab+nivolumab therapy [30].

Monitoring of these different populations could potentially be helpful to predict clinical response to anti-ICPi therapies.

- **TCR diversity**

Data on the impact of baseline blood TCR diversity on anti-ICPi efficacy are scarce, based on small cohorts and not yet consistent. In metastatic urothelial cancers, pretreatment peripheral blood TCR clonality below the median was associated with better response to anti-ICPi treatments but did not correlate with mutation load [182]. In anti-PD-1/L1 treated patients a high baseline peripheral TCR diversity correlated with improved survival in urothelial cancer [183] but not in melanoma [184]. Also, in anti-CTLA-4 treated melanoma patients, one study reported that high baseline TCR diversity correlated with clinical benefit (n=12) [185]) but not the other (n=21) [186] suggesting the need of larger cohorts to confirm this.

Moreover, immunotherapeutic interventions can alter the blood TCR repertoire. Importantly, increased TCR repertoire diversity is induced upon CTLA-4 blockade in blood of patients with melanoma and prostate cancer [186, 187] and associated with improved clinical outcomes [187].

### Myeloid cells and other circulating biomarkers

- **NLR**

A recent meta-analysis in 738 patients [188] reported that high baseline NLR is associated with a reduced response rate to ICPi treatment (ipilimumab, nivolumab or combination) in several solid malignancies (melanoma, non small cell lung carcinoma  (NSCLC), genitourinary cancer). Moreover, high post-treatment NLR correlated with an increased risk of relapse or death in multivariate analysis in anti-PD-1-treated NSCLC patients [189–191]. The addition of NLR to the previously described prognostic score based on objective variables (LDH, serum albumin, number of metastatic sites) might help to better select patients for immunotherapy phase-I trials.

- **MDSC**

The number of immunosuppressive MDSC has been found to negatively affect incidence of response to anti-CTLA-4 treatment and patient OS [166, 168, 192]. In contrast, conflicting data exist for their impact on anti-PD-1 efficacy with negative correlation with OS [193] or no impact [194]. In contrast in this latter study, CD14^+^CD16^neg^HLA-DR^high^ classical monocytes counts were strongly correlated with good outcome.

- **Eosinophils**

Eosinophils have important functions for tumor surveillance and were described as effectors for tumor rejection in animal models [195]. In patients treated with ipilimumab, high baseline absolute eosinophil count correlated with improved OS [166, 196] and early increase of eosinophil counts during treatment was associated with improved clinical responses [165, 192, 196, 197]. Similar observations were recently reported for treatment with anti-PD-1 in melanoma patients [168] but not in NSCLC patients [190].

- **Seric soluble parameters**

Soluble serum biomarkers might also correlate with clinical benefit of ICPi treatment. High levels of the soluble form of PD-L1 (sPD-L1) in plasma represent a poor prognosis factor in various solid tumors (RCC, HCC, NSCLC, gastric cancer and B cell lymphoma) suggesting either a high tumor burden or that sPDL-1 interact with PD-1 and inhibits T cell function. Moreover, high pre-treatment levels of sPDL-1 were associated with increased likelihood of progressive disease in melanoma patients treated by CTLA-4 or PD-1 blockade [198]. Furthermore, detection of soluble ligands of NKG2D (sNKG2DL) in the serum of cancer patients was inversely correlated with NK and T cell activity although they were also correlated to advanced stages of the disease with an overall negative impact on patient survival [199, 200]. In melanoma patients undergoing anti-CTLA-4 or anti-PD-1 mAb therapy, the absence of sMICA and sMICB in post-treatment serum was significantly associated with improved OS [200]. Furthermore, high pre-treatment serum levels of sCD25 were shown to be associated with shorter OS in anti-CTLA-4-treated patients [201].

### Correlation between blood and tumor biomarkers

Several biomarkers of response to ICPi blockade have been characterized in the tumor environment (for review [163]). The tumor mutation burden, microsatellite instability, defects in DNA repair machinery are indicative of hypermutation that would increase neo-antigens and neo-antigen density predict response to ICPi blockade. Moreover, intratumoral biomarkers indicative of an inflamed phenotype such as i) PD-L1 expression, ii) inflammatory gene signature, iii) T cell infiltration, iv) TCR diversity and v) cytokine production have also been related to ICPi blockade efficacy. Finally, the microbiome and the germline genetics characterizing the host environment beyond the tumor microenvironment also impact on efficacy of ICPi.

A limited number of publications correlate peripheral blood biomarkers with tumor biomarkers and response to ICPi treatment. Huang et al recently developed a “reinvigoration score” by relating changes in circulating exhausted CD8^+^ T cells to tumor burden that predicts anti-PD-1 response [202] that could be partly explained by TCR clonotypes shared by exhausted-blood CD8^+^ T cells and TILs, although immune cell functions within the tumor environment markedly differ from those in blood. Another study [203] reported an association between a high circulating CD4^+^ and CD8^+^ T_CM_/T_Eff_ ratio and tumor inflammation in melanoma and NSCLC patients, as well as increased PDL-1 expression in the tumor and longer PFS in response to nivolumab treatment in NSCLC patients.

## Combined therapies to increase ICPi treatment efficacy

Overall, in lymphopenic patients, the recovery of normal ALC is associated with anti-ICPi responses. Besides the current strategies exploring combination of different ICPi antagonists or with ICP activator agonists (OX40, 4-1BB, ICOS), approaches aiming to increase the T cell pool and its diversity should be considered.

### Combination with chemotherapy

An ongoing clinical trial from our institution (NCT03139851) is currently investigating, in lymphopenic patients with metastatic BC, the combination of metronomic doses of cyclophosphamide and anti-PD-1 with the objective to avoid chemotherapy-induced lymphodepletion, reduce the number and functionality of immunosuppressive Tregs [204], favor the reactivation of existing tumor specific T cells with anti-PD-1, and possibly increase the total T cell number and their TCR diversity.

### Combination with radiotherapy

Recent experiments suggest that at least one mechanism of radiation-induced tumor control is the abscopal effect involving the stimulation of the adaptive immune system by tumor neo-antigen release following RT (for review [95]). Robust preclinical data support the combination of SBRT, that reduces the risk of lymphopenia [97, 98], with ICPi blockade administered simultaneously or sequentially in order to stimulate the adaptive immune system, with further amplification by systemic ICPi therapy [205–208]. The few clinical data available on this topic combining anti-CTLA-4 treatment with RT suggest better outcomes [209]. However, tumors associated with a high inflammatory state might be excluded from these combined strategies because of increased risk of severe RT-induced lymphopenia [99]. RT delivery prior to ICPi may release antigens, recruit T-cells and help to increase TCR repertoire diversity, this phenomenon being amplified by ICPi treatment.

### Combination with TIL therapy

In contrast to the negative role of lymphopenia on efficacy of ICPi blockade, efficacy of TIL therapy is improved by prior lymphodepletion [46]. This lymphodepletion is thought to improve the effector function of TILs by i) depleting endogenous Tregs, ii) reducing the number of endogenous lymphocytes competing with the transferred TILs for use of IL-7 and IL-15 produced by non-lymphoid sources in response to lymphopenia [210] and iii) generating “physical space” for the infusion product. TILs may also be used in combination with ICPi to overcome the existing lymphopenia. The feasibility of combined treatment with ipilimumab and standard TIL therapy was recently demonstrated in a small cohort of metastatic melanoma patients [211] with response rates (38.5%) similar to those observed in other TIL trials.

### Combination with cytokines

*In vitro* culture of purified human T cells with recombinant hIL-2 and hIL-15 strongly enhances the expression of PD-1 whereas it remains mild in presence of rhIL-7 [138, 212]. In agreement, in murine tumor models, IL-7 reduces PD-1 expression on activated CD8^+^ T cells favoring their revitalization [152]. In this context, these cytokines are currently evaluated in combination with anti-ICPi therapies.

Therapeutic strategies combining high dose of rhIL-2 (HD IL-2) and anti-PD-1 are being evaluated in clinical trials in melanoma (NCT03476174) and RCC (NCT02964078, NCT02989714). Even if recent data support the efficacy of HD IL-2 [213], its use may favor development of toxicities but also favors the expansion of T cell subsets other than Tregs. The replacement by IL-2v could circumvent both toxic effects and Tregs expansion due to its poor binding on IL-2Rα. Moreover, immuno-cytokines such as anti-CEA-IL-2v will target the delivery of IL-2v within the tumor environment expressing CEA antigen therefore reducing its systemic toxicity and favoring T cell expansion at the site of tumor response. Preclinical results in the MC38 model demonstrate that CEA-IL2v combined with anti-PD-L1 therapy yields greater antitumor efficacy than the respective monotherapies or the combination with an untargeted control immuno-cytokine [214].

IL-15 is currently being evaluated in combination with anti-CTLA-4 or anti-PD-1 antibodies or their combination in refractory solid tumors (NCT03388632). In parallel, IL-15 super agonist (ALT-603) combined with anti-PD-1 therapy is being assessed in metastatic non-small cell lung cancers (NCT0322866). No efficacy result has been published so far.

For IL-7, despite promising clinical impact in a randomized study [160], no clinical trial is investigating combination of anti-ICPi therapy and IL-7. Of importance, recent data in murine bladder tumors report that efficacy of CTLA-4 and PD-1 blockade combination relies on the interdependence between IL-7 and IFNγ signaling in T cells as the lack of either pathway abrogates therapeutic effects [84].

The scientific community is hoping a solution will be found to evaluate the promising combination between ICPi and IL-7.

## Conclusion

As reported in this review, the immune system is quantitatively and qualitatively altered in a multimodal manner in cancer patients in the tumor environment itself as well as in the peripheral blood favoring the evasion of tumor cells from the immune system. Peripheral lymphopenia, pre-existing or induced by therapies in more than 20% of patients with metastatic solid tumors strongly impacts their survival. Although CD4^+^ lymphopenia is a powerful marker of reduced survival, its causes remain uncertain and need to be better investigated (tumor or host origin, inter-relation with TILs,…). Despite its major impact on survival, lymphopenia has not routinely been used to select candidates in anti-CTLA-4 and PD-1 immunotherapy protocols until recently. This emphasizes the importance in future immunotherapy clinical trials to analyze the combination of CD4 lymphopenia, TCR diversity and intensity of the T cell response. This also highlights the medical need for new therapies to restore T cell numbers and diversity and the urgency to find solutions for the clinical development of IL-7 allowing to regenerate thymic functions.
